# Contested psychiatric ontology and feminist critique: ‘Female Sexual Dysfunction’ and the *Diagnostic and Statistical Manual*

**DOI:** 10.1177/0952695112456949

**Published:** 2012-10

**Authors:** Katherine Angel

**Affiliations:** University of Warwick, UK

**Keywords:** American Psychiatric Association, *Diagnostic and Statistical Manual*, Female Sexual Dysfunction, feminism and post-feminism, psychiatric ontologies

## Abstract

In this article I discuss the emergence of Female Sexual Dysfunction (FSD) within American psychiatry and beyond in the postwar period, setting out what I believe to be important and suggestive questions neglected in existing scholarship. Tracing the nomenclature within successive editions of the American Psychiatric Association’s *Diagnostic and Statistical Manual *(*DSM*), I consider the reification of the term ‘FSD’, and the activism and scholarship that the rise of the category has occasioned. I suggest that analysis of FSD benefits from scrutiny of a wider range of sources (especially since the popular and scientific cross-pollinate). I explore the multiplicity of FSD that emerges when one examines this wider range, but I also underscore a reinscribing of anxieties about psychogenic aetiologies. I then argue that what makes the FSD case additionally interesting, over and above other conditions with a contested status, is the historically complex relationship between psychiatry and feminism that is at work in contemporary debates. I suggest that existing literature on FSD has not yet posed some of the most important and salient questions at stake in writing about women’s sexual problems in this period, and can only do this when the relationship between ‘second-wave’ feminism, ‘post-feminism’, psychiatry and psychoanalysis becomes part of the terrain to be analysed, rather than the medium through which analysis is conducted.

## Introduction

‘Female Sexual Dysfunction’ (FSD) is a term that has gained in medical, psychiatric and media prominence in recent decades, especially in the last 15 years. It is not in itself a diagnostic category, but has gained a currency that can sometimes suggest otherwise. Female sexual problems are currently classified in the American Psychiatric Association’s 4th edition of the *Diagnostic and Statistical Manual* (*DSM*) (American Psychiatric Association, 2000), in a section on Sexual Dysfunctions (separate both from the paraphilias and from gender identity disorders), and assembling diagnoses relating to desire, arousal, orgasm and pain (see table 1). The *DSM*’s rise has been much criticized, and while the growing discussions of FSD are praised by researchers and scientists as an index of a long-overdue attention to women’s sexuality – a triumph of feminism (Basson *et al.*, 2001; Berman and Berman, 2001) – critics have underscored the link between this renewed medical attention and pharmaceutical interests in launching a compound comparable to Viagra for ‘Erectile Dysfunction’ (Tiefer, 2008; Kaschak and Tiefer, 2001; Loe, 2004).

**Table 1.** *DSM*-IV, 1994.

SEXUAL DISORDERS	
Paraphilias	Sexual Dysfunctions
Exhibitionism	Hypoactive sexual desire disorder
Fetishism	Sexual aversion disorder
Frotteurism	Female sexual arousal disorder
Pedophilia	Male erectile disorder
Sexual masochism	Female orgasmic disorder
Sexual sadism	Male orgasmic disorder
Transvestic fetishism	Premature ejaculation
Voyeurism	Dyspareunia
Paraphilia not otherwise specified	Vaginismus
	Sexual dysfunction due to a general medical condition
	Substance-induced sexual dysfunction

The FSD debate plays itself out in the public and popular domains as well as in the medical, psychiatric and sexological realms. Existing historical scholarship on female sexuality in the postwar period focuses primarily on sexology (e.g. Gerhard, 2001; Irvine, 1990). In this article, I suggest that analysis of FSD benefits both from scrutiny of a wider range of sources (especially since the popular and scientific cross-pollinate), and from an approach from the history of psychiatry, in particular one that probes debates about the ontology and aetiology of sexual disorders, and the relationship between feminism and psychiatry in the postwar period. I suggest that existing literature on FSD has not yet posed some of the most important and salient questions at stake in writing about women’s sexual problems in this period, and can only do this when the relationship between 'second-wave' feminism, 'post-feminism', psychiatry and psychoanalysis becomes part of the terrain to be analysed, rather than the medium through which analysis is conducted.

I begin by outlining a brief sketch of female sexual problems in the 20th century, and their rendering in the first two postwar *DSM*s. I then give an account of the emergence of *DSM*-III in 1980, and the classifications of sexual disorders in this manual. I then discuss both the reification of ‘FSD’ as a term, in relation to Viagra’s emergence in the late 1990s, and the recent activism and scholarship around FSD. I explore the multiplicity of FSD that emerges when one examines a wider range of sources, but also underscore a reinscribing of anxieties about psychogenic aetiologies. I then argue that what makes the FSD case additionally interesting, over and above other conditions with a contested status, is the historically complex relationship between psychiatry and feminism that is at work in contemporary debates. I conclude with some thoughts about ‘post-feminism’ and the historiographical challenges raised by FSD.

## Sexual problems and the *DSM*

Women’s sexual problems have been written about prolifically during the 20th century, in sexological, gynecological, psychiatric and psychoanalytic literature, as well as marital advice material (e.g. Ellis, 1897–1928; Krafft-Ebing, 1899; Hitschmann and Bergler, 1936; Stekel, 1936; Stopes, 1918; Wright, 1955[1930]; Van de Velde, 1928[1926]; Gray, 1923; see Lunbeck, 1994; Downing, 2004; Irvine, 1990; Maines, 1999; Cook, 2004; Cryle, 2009; Angel, 2010). Psychiatry has been salient in these discussions due to the mutually entangled development of psychiatry, sexology and criminology in the last quarter of the 19th century (Foucault, 1979; Bland and Doan, 1998; Davidson, 2001). A forensic rationale fostered a focus in late 19th- and early 20th-century sexology on sexual behaviours and identities, with the reification of classifications such as homosexuality, sadism, masochism and fetishism. Frigidity – and its flip-side, nymphomania – were much invoked preoccupations in the first half of the 20th century, enduring until the 1960s at least; other concepts throughout the century have included sexual anaesthesia, sexual inhibition, anorgasmia, or preorgasmia, and, from the 1970s, sexual dysfunction. Frigidity especially has been a semantically shifting term variously meaning women’s natural lack of desire, their unnatural failure to experience a normal desire, their failure to become aroused, failure to reach orgasm, or failure to reach *vaginal *orgasm (Loewenfeld, 1899; Acton, 1862, quoted in Ellis, 1908: 157–8; Hitschmann and Bergler, 1936; Stekel, 1936; Huhner, 1937). A vast literature has documented the neo-Freudian linking of a range of social and psychological ills to a clitoral, rather than a vaginal, sexuality, where the outlining of norms for female sexuality has functioned as a way of defining norms of femininity and heterosexuality (e.g. in Abraham, 1920; Bonaparte, 1953; Deutsch, 1944–5; see Buhle, 1998; Lunbeck, 1994; Irvine, 1990; Maines, 1999; Downing, 2004).

In the postwar period, the American Psychiatric Association (APA) and its *DSM* loom increasingly large in psychiatry and in matters sexual. In its 1st edition in 1952 (an attempt to consolidate the many local variations to the American Medico-Psychological Association’s existing nomenclature and a proliferation of military nomenclatures), problems such as impotence and frigidity are an instance of ‘Psychophysiological autonomic and visceral disorders’ (under a larger group of ‘Disorders of psychogenic origin or without clearly defined physical cause or structural change in the brain’), of which a ‘psychophysiological genito-urinary reaction’ is an instance. We are told that these include ‘some types of menstrual disturbance, dysuria, and so forth, in which emotional factors play a causative role’ (American Psychiatric Association, 1952: 30). (See table 2.)

**Table 2.** *DSM*-I, 1952.

Personality disorders	Disorders of psychogenic origin or without clearly defined physical cause or structural change in the brain
Sociopathic personality disturbance	Psychophysiological autonomic and visceral disorders
Sexual deviation	Psychophysiological genito-urinary reaction
e.g. homosexuality transvestism pedophilia fetishism sexual sadism	e.g. ‘some types of menstrual disturbance, dysuria and so forth, in which emotional factors play a causative role’

These disorders represent the visceral expression of affect that is often ‘prevented from being conscious’. Symptoms are due to a ‘chronic and exaggerated state of the normal physiological expression of emotion, with the feeling, or subjective part, repressed. Such long continued visceral states may eventually lead to structural change’ (American Psychiatric Association, 1952: 29).

In the 1968 *DSM*-II (see table 3), we see a slight variation of this; ‘psychophysiological disorders’ include ‘genito-urinary disorders such as disturbances in menstruation and micturition, dyspareunia, and impotence in which emotional factors play a causative role’ (American Psychiatric Association, 1968: 47). These ‘psychophysiological disorders’ are characterized by

… physical symptoms that are caused by emotional factors and involve a single organ system, usually under autonomic nervous system innervation. The physiological changes involved are those that normally accompany certain emotional states, but in these disorders the changes are more intense and sustained. The individual may not be consciously aware of his emotional state. (American Psychiatric Association, 1968: 46)

Little detail is given, then, of the specific kinds of disorders included in these categories (in *DSM*-I, readers are told that each diagnosis of ‘psychophysiologic autonomic and visceral disorders’ will be ‘amplified with the specific symptomatic manifestations, e.g. anorexia, loss of weight, dysmenorrhea, hypertensions, and so forth’ (American Psychiatric Association, 1952: 29), and the kinds of disorders enumerated are not meant to be comprehensive. An aetiological process is posited, and it is the *process* – rather than its specific manifestations – that is salient. In their focus on unconscious, repressed emotions, the manuals’ terminology reflects the importance within American psychiatry, between the 1930s and 1960s, of an American-inflected psychoanalysis (Hale, 1995); in their emphasis on physiological changes that accompany certain emotions, and eventually, if sustained for long enough, lead to structural damage, they reflect the influence of a psychosomatic medicine much shaped by Franz Alexander (Alexander, 1932, 1950; Brown, 2000; Greco, 1998). Moreover, the manuals, in their emphasis on mental illness as on a continuum with health, also voice the influence of Adolf Meyer’s psychobiology, in which environmental factors interacted with certain predisposing factors in an individual to determine his or her capacity to adapt to that environment (Meyer, 1952). The *DSM*-III of 1980, however, ushers in significant changes in classification, marking an important shift in psychiatry’s 20th-century history.

**Table 3.** *DSM*-II, 1968.

Personality disorders and certain other non-psychotic mental disorders	Psychophysiological disorders
Sexual deviations	Psychophysiological disorders (physical disorders of presumably psychogenic origin)
e.g. homosexuality, fetishism, pedophilia, transvestism, exhibitionism …	Psychophysiological genito-urinary disorders‘such as disturbances in menstruation and micturition, dyspareunia, and impotence in which emotional factors play a causative role’

**Table 4.** *DSM*-III, 1980.

	PSYCHOSEXUAL DISORDERS	
Gender Identity Disorders	Paraphilias	Psychosexual Dysfunctions
Transexualism	Fetishism	Inhibited sexual desire
Gender identity disorder of childhood	Transvestism	Inhibited sexual excitement
	Zoophilia	Inhibited female orgasm
	Pedophilia	Inhibited male orgasm
	Exhibitionism	Premature ejaculation
	Voyeurism	Functional dyspareunia
	Sexual masochism	Functional vaginismus
	Sexual sadism	Atypical psychosexual dysfunction
	Atypical paraphilia	

## *DSM*-III

The *DSM*-III of 1980 emerged out of a period of intense controversy and questioning in the psychiatric profession and beyond. Psychoanalysis was at the core of American psychiatry by the 1950s, but increasingly competing with other emerging forms of psychotherapy, and increasingly challenged by a critique of its scientific validity made by academic psychologists, behaviourists and philosophers who questioned the very possibility of its generating verifiable predictions and testable theories (see Eysenck, 1959; Eysenck, 1965(a), 1965(b); Popper, 1963; Hook, 1959; Fisher and Greenberg, 1977; Freedheim, 1992; Luborsky, 1975; Wallerstein, 1966; see Hale, 1995: ch. 17). In addition, insurers were becoming unwilling to fund long psychoanalytic treatments whose efficacy was under question (Mayes and Horwitz, 2005). And the critique of psychoanalysis overlapped with some strands of the ‘anti-psychiatry’ movement, which challenged what it saw as diagnostic promiscuity and abusive and authoritarian relationships to the patient – with some critiques focusing particularly on the gender biases at work in psychoanalysis^1^ (Szasz, 1962; Laing, 1960, 1964; Scheff, 1966; Goffman, 1961; Becker, 1963; Sulloway, 1979; Roazen, 1976; Brome, 1967; Medawar, 1975; Gelfman, 1969; Fliegel, 1973; Gilman, 1971, 1977; Friedan, 1963; Chesler, 1972; Millett, 1970; see Grob, 1991; Hale, 1995; Shorter, 1997; Healy, 2002, 1997; Dain, 1989; Nye, 2003; Osborne, 1992; Appignanesi and Forrester, 2000; Tomes, 1994).

This scientific and cultural dissatisfaction was reflected in professional concern over diagnostic confusion and classificatory unreliability, with stark disparities in diagnostic trends causing embarrassment (see Stengel, 1959; Kramer, 1969; Kendell *et al.*, 1971; WHO, 1973). In the late 1970s at the APA, Robert Spitzer, trained in both medicine and psychoanalysis, was appointed to the new task force to revise the *DSM*. He and his collaborators were committed to several axioms about psychiatry and mental illness that overturned the key assumptions of previous *DSM*s; namely, that there is a boundary between the normal and the sick; that there are discrete mental illnesses; and that the focus ‘of psychiatric physicians should be particularly on the biological aspects of mental illness’ (Klerman, 1978: 104, see Bayer and Spitzer, 1985). The *DSM*-III sought to remove aetiological assumptions (of a psychoanalytic kind, emphasizing neurosis and defences) that it argued were unsupported. Disorders were discrete, and operationalized reliably by sets of symptom criteria (Rogler, 1997); an effect, argues Healy, of the rise of the randomized, placebo-controlled trial to test new medications required by the FDA provably to target clearly definable illness (see Healy, 1997, 2002; Pignarre, 2004). Highly controversial at the time for its excision of psychoanalytic conceptions, the *DSM*-III has consistently provoked controversy since, though latterly focused on the *DSM*’s expansion and its role in providing a rationale for pharmaceutical products (Mayes and Horwitz, 2005; Horwitz and Wakefield, 2007; Kirk and Kutchins, 1992; Kutchins and Kirk, 1997; Angell, 2011; Watters, 2010; Lane, 2007; Lakoff, 2005; Moynihan and Mintzes, 2010). For good or for ill, the *DSM *is now ‘connective tissue for biomedical psychiatry’, binding together measurement tools, diagnosis, treatment, research, drug assessment, epidemiology, insurance, and the law (Lakoff, 2005: 13).

## The *DSM*-III and sexual dysfunctions

*DSM*-III yielded an increasingly detailed and differentiated classification of disorders, subsumed under a new splitting of diagnostic areas (e.g. ‘Neurotic Disorders’ were dispensed with, but symptomatic remnants were distributed into the affective, anxiety, somatoform and dissociative disorders) (see Rogler, 1997). The manual reorganizes the two categories of sexual problems that existed in *DSM*-I and *DSM*-II; instead of entirely separate categories and chapters for ‘Sexual Deviations’ and ‘Psychophysiological Genito-urinary Disorders’, in *DSM*-III we have an overarching chapter on ‘Psychosexual Disorders’. (See table 4.)

This is broken down into gender identity disorders, paraphilias and psychosexual dysfunctions (with the latter using gender as a defining principle). The revised *DSM*-III-R of 1987 changed ‘Psychosexual Dysfunctions’ to ‘Sexual Dysfunctions’, and lists the various disorders under the overarching categories of ‘Sexual Desire Disorders’, ‘Sexual Arousal Disorders’, ‘Orgasm Disorders’ and ‘Sexual Pain Disorders’, among other changes (see table 5). *DSM-*IV (1994) remains much the same, except that ‘Inhibited female orgasm’ becomes ‘Female orgasmic disorder’. (See table 1.)^2^

**Table 5.** *DSM*-III-R, 1987.

SEXUAL DISORDERS	
Paraphilias	Sexual Dysfunctions
Exhibitionism	Hypoactive sexual desire disorder
Fetishism	Sexual aversion disorder
Frotteurism	Female sexual arousal disorder
Pedophilia	Male erectile disorder
Sexual masochism	Inhibited female orgasm
Sexual sadism	Inhibited male orgasm
Transvestic fetishism	Premature ejaculation
Voyeurism	Dyspareunia
Paraphilia not otherwise specified	Vaginismus
	Sexual dysfunction not otherwise specified

Note: Transexualism and Gender Identity Disorder are moved in *DSM-III-R* to a separate category of ‘Disorders Usually First Evident in Infancy, Childhood, or Adolescence’.

The classification, since *DSM*-III, is much indebted to the work of Masters and Johnson, whose *Human Sexual Response *and *Human Sexual Inadequacy *were published in 1966 and 1970 respectively. Masters and Johnson were not the first to approach sexual problems with a form of behavioural conditioning, or to emphasize the clitoris in female sexual pleasure (Rachman, 1961; Lazarus, 1963; Kinsey *et al.*, 1953; see also Downing, 2004). But their detailed physiological studies heralded the professionalization of a behaviouristically inflected sex therapy. Concerned to draw a near-total equivalence between male and female sexual function,^3^ they outlined a four-stage ‘Human Sexual Response Cycle’ (excitement, plateau, orgasm, resolution), and three female sexual disorders: dyspareunia, vaginismus and orgasmic dysfunction (primary and secondary) (Masters and Johnson, 1966, 1970). (The problems for men were premature ejaculation, ejaculatory incompetence and primary and secondary impotence.)

Masters and Johnson’s ideas and nomenclature were mediated through Helen Singer Kaplan, who was a member of the Sexual Disorders Committee for *DSM*-III, and a sex therapist and psychoanalyst who sought to combine behaviourist and psychoanalytic models in her work. She modified their nomenclature into a three-stage model of desire, excitement and orgasm, incorporated into the document (American Psychiatric Association, 1980; Tiefer, 2004: 51–2). Together with psychoanalyst Harold Lief, who also served on the *DSM*-III Sexual Disorders Committee, she ensured that problems of desire, as well as those of arousal and orgasm, were foregrounded (see Kaplan, 1977 and 1979; Lief, 1977). APA archival material, however, also reveals that psychoanalyst Robert Stoller, with whom Spitzer kept up a detailed correspondence, similarly (and earlier) suggested abandoning Masters and Johnson’s distinction between the excitement and plateau phases:

There is one issue with which I am out of step as compared to other colleagues in sex research. I am not sure there is good reason to separate out an excitement phase from a plateau phase. I do not believe that what is called ‘plateau’ is anything other than excitement. It is as if we were to divide excitement into a number of non-distinguishable episodes; such distinctions have no real function. (Stoller, 1977)

This renewed emphasis on desire – problems of which had been a significant component of the neo-Freudian writings on frigidity – undid an emphasis on the mechanics of arousal and orgasm in Masters and Johnson. Kaplan’s categories of ‘inhibited’ desire, excitement and orgasm reinscribed a psychoanalytic affinity, with the concept of inhibition containing within it the psychoanalytic connotations of symptoms resulting from the defences mounted against anxiety. So while criticisms of the *DSM*’s FSD nomenclature have tended to focus on their allegedly uncritical adoption of a flawed and mechanistic Masters and Johnson model (Tiefer, 2004), and criticisms of the methods and rationale of the *DSM*-III ‘revolution’ have underlined Spitzer’s ruthless eviction of psychoanalysis from the manual (and the profession) (Kutchins and Kirk, 1997), it appears that, at least for the Sexual Disorders, this narrative of eviction does not hold up so well, suggesting that a vision of the move to *DSM*-III as involving a stark and wholesale shift away from psychoanalytic concepts is in need of some refinement.

## ‘Female Sexual Dysfunction’ and ‘Erectile Dysfunction’

In Masters and Johnson’s books, the term ‘female sexual dysfunction’ operated simply as a generic description rather than as a specific diagnosis; it signified any problem with sexual function as identified in the human sexual response cycle. But the term ‘Female Sexual Dysfunction’ becomes more prominent after their publications, however. It begins to appear in medical and scientific journals as a phrase only from 1977 onwards; half a dozen results appear in the 1970s, and the same number in the 1980s – a period when the term ‘frigidity’ is waning in frequency. Occurrences of ‘FSD’ from the 1990s onwards jump to being in the 500s. And yet there is in fact no such term ‘FSD’ in the *DSM*s; the chapter headings are ‘Psychosexual Disorders’ (*DSM*-III) or ‘Sexual Disorders’ (*DSM*-III-R), under which the various subtypes appear.

This jump in FSD discourse occurs around the time of Viagra’s licensing for Erectile Dysfunction (ED) in 1998. Viagra’s apparently phenomenal success, and its astounding sales, put a premium on finding a similar drug for women. Pharmaceutical companies rushed to test Viagra in women and sought to develop other compounds (Loe, 2004; Hartley, 2003, 2006). What we see, from the 1990s onwards, is a significant slippage between ‘FSD’ as a generic umbrella term – where the general type of dysfunctions is sexual, and the ‘female’ works as a qualifier – to ‘FSD’ figuring as a condition in itself, a slippage enabled by the use of the singular term ‘dysfunction’. An example is a 1999 article by Jennifer and Laura Berman and Irwin Goldstein (prominent and prolific authors – the former in scientific and popular texts – on ED and FSD), which opens thus: ‘Female sexual dysfunction is age-related, progressive, and highly prevalent, affecting 30% to 50% of women’ (Berman *et al.*, 1999a: 385).

While they go on to specify particular areas of diagnosis such as ‘vaginal lubrication, pain and discomfort with intercourse, decreased arousal, and difficulty achieving orgasm’, they also state that ‘our knowledge and understanding of the anatomy and physiology of the female sexual response and the pathophysiology of female sexual dysfunction is limited’. And yet, some of the diversity of sexual problems having been roll-called, the slippage to the phrase ‘pathophysiology of female sexual dysfunction’ assumes that the pathway to these diverse phenomena is one and the same, though it remains to be clearly elucidated. In other words, it casts ‘female sexual dysfunction’ as a condition in itself. This pattern is much repeated elsewhere, and articles frequently use the term ‘FSD’ in the title, but quickly move in the text to specifying disorders under consideration (O’Donohue *et al.*, 1997; Sarwer and Durlak, 1996). A recent example is Figueroa-Haas (2012), who uses ‘Female Sexual Dysfunction’ in the title, and writes the following:

Female sexual dysfunction (FSD) occurs when a woman is unable to fully experience pleasure during sexual activity. This dysfunction can affect a woman’s quality of life and lead to personal distress and interpersonal impairments. It is defined as disorders of sexual desire, arousal, orgasm, and sexual pain. An estimated 40% of women are affected by sexual dysfunction and 1 in 4 is unable to achieve orgasm. (Figueroa-Haas, 2012: 156)

In this particularly confused example, FSD is cast as a disorder in itself, offering a *definition *of ‘FSD’ as ‘disorders of desire, arousal’ and so on – whereas these categories are not *definitions *of a disorder, but the diagnostic categories subsumed under a generic term for them. Moreover, it emphasizes pleasure, which potentially brackets out desire problems.

What we are seeing here is FSD being used as an analogue – a counterpart – to ED, as in an article entitled ‘Lifestyle/Dietary Recommendations for Erectile Dysfunction and Female Sexual Dysfunction’ (Esposito and Giugliano, 2011). But ED is *one* diagnostic category applicable to men in the ‘Sexual Disorders’ section of the *DSM*, while FSD is not its equivalent – it is a generic umbrella term for diagnoses that vary significantly. This reification of ‘FSD’ both reflects and bolsters the desire to see ‘FSD’ as a discrete, bounded diagnostic category amenable to a technology that would target it in the allegedly specific way Viagra did for ED – a desire that nonetheless has yielded little in the way of concrete results.^4^

## Activism and scholarship

The rise of ‘FSD’ and pharmaceutical efforts around it has provoked considerable controversy as well as activism. New York sexologist Leonore Tiefer founded the New View Campaign, ‘challenging the medicalisation of sex’ (see Kaschak and Tiefer, 2001), after the increase, post-Viagra, in the ‘marketing’ of FSD, having previously written about ED (Tiefer, 1994, 2004). The campaign sees Viagra as reinforcing a mechanical view of sexuality, rendering penetration and orgasm the pinnacle of sexual activity, and expressing hetero-normative ideals of sexual behaviour. It argues that FSD discourse reduces sexuality to the purely biological as malfunctions of vascular, hormonal, or cerebral systems to be fixed by pharmacological intervention, suggesting instead that female sexual problems especially are highly contextual – the result of social, cultural and political factors (lack of communication between partners; exhaustion due to inequalities in child-rearing and housework; anxieties about body image; violence from sexual partners; and misunderstandings of female anatomy; [see http://www.newviewcampaign.org/manifesto.asp]). The campaign has received growing press coverage in the last few years (Vernon, 2010; Sample, 2009; *Independent*, 2010a; Laurence, 2010; *Independent*, 2010b).

The campaign is also interlinked with a growing scholarship on FSD, the *DSM* and pharmaceutical developments for sexual problems (much coming from social science, qualitative research and science studies), part of a larger movement challenging the ‘medicalization’ of problems seen as social, political, contextual and relational in nature (Cacchioni, 2007; see Potts, 2000; Potts *et al.*, 2004; Potts *et al.*, 2003 for material on masculinity and Viagra; Ramage, 2006a, 2006b; Fishman, 2004; Nicolson and Burr, 2003; Kaschak and Tiefer, 2001; Potts, 2008). Polemical and general audience works are also interlinked with the campaign (Drew, 2003; Moynihan and Cassels, 2005; Moynihan and Mintzes, 2010).

Many of these works owe much in spirit to the discourses of ‘anti-medicalization’ that have been such a prominent part of debates about medicine and psychiatry since the 1960s. And they owe much to a component of second-wave feminism, suspicious of psychiatric expertise and professional expansion of authority over women’s health, bodies and minds (e.g. ‘Towards a Feminist Sex Therapy’, in Tiefer, 2004).

That the existing scholarship takes its impetus largely from activist concerns has some important methodological consequences. It necessitates and explains a focus on the *DSM* and on pharmaceutical developments. But in consequence, it narrowly construes the FSD landscape, glossing over resources such as women’s magazines and self-help books, for instance, which speak in an emotional, psychological *and *behavioural register. Moreover, it also glosses over the multiplicity at work in medical texts themselves.

## Multiplicity

Take, for example, the pages of *Cosmopolitan. S*ex psychotherapist Rachel Morris responds to a letter about libido problems:

‘A lack of desire can be due to many things’, hormone imbalance included, … but usually it’s down to a psychological or emotional block. There are so many reasons to freak out about sex – what if I’m no good? What if I don’t orgasm? It could be that you let negative thoughts freeze desire before it starts. … Adding pressure will only make it worse, and stress, depression and anxiety are libido killers. Sexuality can’t be forced – it has a life of its own. Talk to your GP and ask for psychosexual counselling. (*Cosmopolitan*, May 2001: 36)

Similarly, Jennifer and Laura Berman, authors of the best-selling *For Women Only: A Revolutionary Guide to Overcoming Sexual Dysfunction and Reclaiming Your Sex Life* (Berman is also the author of *The Passion Prescription: Ten Weeks to Your Best Sex – Ever!*), and prolific in medical journals also, advocate a significantly medical and pharmacological framework for sexual problems, especially the virtues of Viagra and testosterone; but they too see fantasy, imagination and thoughts as key to ‘helping yourself’ with your sexual problems. In these texts, emotional, behavioural and cognitive habits are on a par with medical drugs, pornography and sex toys; factors key to enhancing pleasure and overcoming sexual problems. Likewise, health information websites such as Netdoctor identify a range of factors in sexual problems (Webber and Delvin, 2011), and a representative article in the *American Family Physician* lists, as possible causes of sexual problems: medicines; diabetes; high blood pressure; alcohol; vaginal infections; menopause; depression, ‘an unhappy relationship or abuse (now or in the past)’; the stresses of everyday life, or being ‘bored by a long-standing sexual routine’ (*American Family Physician*, 2000: 141–2).

The ontology of sexual problems we see in a range of resources is, I think, more cacophonous and multiple than a focus on pharmacological reductionism suggests. It is perhaps an index of a late 20th- and early 21st-century ‘surveillance’ medicine in which multiple symptoms and signs become risk factors for future outcomes (Armstrong, 1995; Rose, 2007). And yet, while a great many things – hormones and emotional blocks, as *Cosmopolitan* tells us – are relevant to sexual problems, they are not all equal.

A kind of ontological hierarchy is asserted in a range of texts, with those construed as psychosomatic, psychological, or psychodynamic – as partaking of the ‘unscientific’ psychiatry which *DSM*-III overturned – figuring as morally and scientifically fraught. A 2001 Consensus Report written by prominent urologists and psychiatrists, for example, proposes a nomenclature that would cover both ‘psychogenic *and* organically based disorders’ (unlike the *DSM*, which it describes as narrowly psychiatric, ‘not intended to be used for classification of organic causes of female sexual dysfunctions’; Basson *et al.*, 2001: 92). This is a curious reading of the *DSM*, given the latter’s insistence on its own nature as providing a medical nomenclature, and as aetiologically neutral (American Psychiatric Association, 1994: xvii–xviii). But it reveals thereby the ambivalence with which the past of psychiatry – with its aetiologies that so prioritized psychogenic factors in causing symptoms – is experienced. Location *in *the *DSM* is problematic, signalling a discomfort with psychiatric theorizing that also sustains the claim that female sexuality, unlike male sexuality, has not, historically, been the subject of medical scrutiny (Basson *et al*., 2001: 83–4) – a claim that overlooks the location of female sexual problems within sexological, psychiatric and psychoanalytic work in the 20th century. As such, it reflects a contemporary discomfort with the psychodynamic past of psychiatry that necessitates defining it, retrospectively, as non-medical. Psychiatry, its own medical protestations notwithstanding, is experienced as a troubling fringe discipline with a troubling past: female sexual problems should be removed ‘from the realm of primarily psychiatric disorders … into the *mainstream* of research and treatment’ (Rosenthal, 2001: 203; emphasis added).

The fraught status of the psychogenic aetiologies associated with a psychiatry that has been left behind is also evident in responses to a *British Medical Journal *article in 2003 written by Ray Moynihan that described the ‘making of’ FSD by researchers with close ties to drug companies (Moynihan, 2003: 45). Outraged responses ensued. A nurse therapist wrote that FSD is not a ‘new, pharmaceutically manufactured condition’ and that it is ‘ignorant in the extreme’ to think that she is ‘only treating “social disorders”’ (Astbury-Ward, 2003). Sexual dysfunction is not, a patient wrote, a ‘fabrication’ of women’s imagination or of ‘corporate America’s financial imagination’ (Dionne, 2003). Another patient wrote that a woman is ‘effectively silenced by the constant response that it is psychological (i.e. that she is imagining it)’ (Lewis, 2003). Similarly, Jennifer and Laura Berman – a urologist and psychiatrist team – write that they are ‘shocked’ to hear of doctors telling patients that their sexual problems are ‘emotional, relational, or due to fatigue from child rearing or their busy jobs’:

We hope that this book will serve as an antidote to what women have heard for decades. The problem is not just in your head. You are not crazy. …We are beginning to recognize female sexual dysfunction as a medical problem. (Berman and Berman, 2001: x–xi)

In the wake of Viagra, much-cited articles state that the context of female sexuality is highly important, but their priority remains to frame FSD as medical rather than psychiatric or psychological, as in Berman *et al.* (1999a: 389), which states that ‘*not all female sexual complaints are psychological, and that there are possible therapeutic options*’. This article, and others, frames the psychological as secondary matter, which rhetorically functions as a concession in the article; they also echo an aspect of the Viagra/ED debate, in which Viagra’s apparent success was invoked (though this is not logically compelling) as proof that ED is ‘physical’, not psychological (Berman *et al.*, 2000; Park *et al.*, 1997; Korenman, 1998; Goldenberg, 1998; Berman *et al.*, 1999a; Berman *et al.*, 1999b; see Loe, 2004; Hartley, 2003).

We are seeing here the slippery power of terms with deep ontological and moral reverberations. Claims about what influences diagnostic categories, aetiological narratives and symptoms themselves are repeatedly interpreted as claims about the *reality* of symptoms. Describing a condition as psychological is interpreted as meaning that it is non-physical; what is not physical is thought to be not real. So saying that something is psychological is equated with saying it is unreal, imaginary, or fake. ‘Psychosomatic illness’, in popular usage, tends to connote deception, if not of others then at least of oneself, raising sensitive questions about wilful motivation and individual culpability (Lawrie, 2000; Stone *et al.*, 2004).

## Psychiatry and feminism

The fraught ontologies in the FSD landscape are, to be sure, not dissimilar from controversies regarding other conditions, Chronic Fatigue Syndrome most notably, where bitter and tail-chasing debates ensue about the ‘reality’ of conditions (Prins *et al.*, 2006). The ontological discomforts I outline here, however, are overlain with something else – the historically uncomfortable relationship between feminism, psychiatry and psychoanalysis, which adds a complex dimension to this landscape.

The fact that FSD scholarship tends to be activist-related explains another phenomenon in the existing literature: a failure to scrutinize, as an object in landscape, the feminism that sustains the critique of it. Feminist critics were vocal in the criticisms of psychiatry, and especially of psychoanalysis, from the 1960s, underscoring gender biases and the violences these were seen to enable (e.g. Friedan, 1963; Chesler, 1972; Millett, 1970; also, in the 1980s, Masson, 1985, 1989). The figure of Freud looms large in these works; Millett described him as ‘beyond question the strongest individual counterrevolutionary force in the ideology of sexual politics’; and Anne Koedt framed the ‘myth of vaginal orgasms’ (1973) as his most pernicious legacy (a legacy repeatedly invoked elsewhere; see Maines, 1999, and a criticism by Lunbeck, 2002). Existing scholarship on female sexuality, psychiatry, medicine and sexology is emphatic in its recognition of the feminist stakes in understandings of female sexual problems (Cook, 2004; Irvine, 1990; Gerhard, 2001). To Irvine, for example, the measure of sexology, and its history, is the extent to which it is compatible with ‘feminism’ (even if the nature of that feminism is not explicitly articulated or spelt out in the book). Indeed, the fields of women’s history, feminist critique and the history of medicine themselves have a thoroughly intertwined, and immensely fruitful, history (Tomes, 1994).

While feminist critiques of FSD emphasize the social, psychological and contextual factors involved in sexual problems, they sidestep the thorny question of psychodynamic understandings of symptoms, or of any potential role of the unconscious in forming symptoms. They articulate a complex position via the nature and causes of sexual problems, but do so under the shadow of the sins of the fathers – the abuses perpetrated in the name of psychiatric and especially psychoanalytic conceptions of sexuality.^5^ As such, it reveals its own historically contingent identity as part of a particular strand of Anglo-American second-wave feminism, evincing an antipathy towards the psychological discourses of sexuality associated with Freud – rather than as part of feminisms that have wrought sympathetic if critical engagement with psychoanalysis (Mitchell, 1974; Chodorow, 1989; Cixous, 1976[1975]; Irigaray, 1977). And while feminist critique of psychoanalysis emerged with good reason, it cannot simply be assumed, theoretically and methodologically, by scholarship that attempts to illuminate the postwar trajectory of female sexual problems within psychiatry and culture – given that the rejection of psychoanalysis by both feminist and psychiatric discourses is such a pivotal phenomenon in that landscape.^6^

In seeking to understand the FSD landscape, I suggest, it is helpful to widen the lens of criticism from discerning the norms of sexuality and gender expressed in explicitly medico-pharmaceutical discourse – vital though that is – to also scrutinizing feminism itself as a category that is discursively managed across a range of realms: medical texts, popular texts, and indeed feminist texts themselves.

Debates about female sexual problems in the postwar period are characterized by ambivalences towards the past, and indeed towards the very act of looking back at the past. Medical, feminist and popular texts evince discomfort with harking back to the past of psychiatric theorizing – which, in psychodynamic psychiatry, involved precisely a commitment to the importance of the past in creating symptoms. Moreover, scholarship on these matters embodies a reluctance to look back, inclusively, at the history of feminism itself: at its complex relationship to psychiatry and its past, and how that shapes the modes in which critique is articulated.

Angela McRobbie has described a post-feminist sexual contract, in which, under the guise of an equality assumed to have been achieved, young women are attributed with political, economic and sexual capacity. ‘Young women are able to come forward’ – as high-achieving, economically powerful figures – ‘on condition that feminism fades away’, and that they abandon the critique of hegemonic masculinity and sexism associated with feminism (McRobbie, 2007: 720, 729). She implies that the rejecting and the historicizing of feminism are essentially interwoven (McRobbie, 2009: 16). We are required to look back at feminism as something unnecessary, old and worn, and the dismantling of feminist politics is, she argues, inextricably linked to the contemporaneous dismantling of feminism within the academy (ibid.: 13).

What relationship, then, does this historical activity – of including feminism as a component in the landscape to be analysed – bear to a ‘post-feminist’ stance that has been diagnosed as a symptom of a contemporary rejection of feminism? How should one look back, historically and critically, at the feminisms of the postwar period? It is my view that an adequate analysis of the landscape requires, in addition to a critical relationship to rejections of feminism, a critical relationship to modes of *rejecting* the activity of looking back at the past. FSD scholarship provides us, I think, with an opportunity – as yet not taken up – to rethink the relationships between ‘second-wave’ feminism, ‘post-feminism’, psychoanalysis, medicine and psychiatry. It provides an opportunity not only to think about the intertwined legacies of psychiatry and feminism, but also to listen to complex discourses about history itself; about what is past, and what is present. It is, in other words, an opportunity to ask, and to find out, what kind of scholarship this particular moment – in psychiatry, feminism and their historiography – is challenging us to write.

## Notes

1. The catch-all term ‘antipsychiatry’ needs to be used with caution; as Colin Jones has underlined, it encompasses a very varied phenomenon made up of sometimes conflicting positions, among which are a content-based critique of psychiatry, a ‘gestural politics of carnivalesque inversion and symbolic performance’ and the exploration of new paradigms of knowledge about what it is to be human; see Jones (1998).

2.
*DSM*-V is, at the time of writing, being prepared, due to be published in 2013; it appears to be significantly reordering the sexual classifications, in particular collapsing desire and arousal problems into a category of Sexual Interest/Arousal Disorder in women, and combining Dsypareunia and Vaginismus into Genito-Pelvic Pain/Penetration Disorder; accessed 21 July 2011 @: http://www.dsm5.org/Pages/Default.aspx

3. A commitment to equivalence has persisted into later *DSM*s, and has been the target of critique (Tiefer, 2004). It is also being rethought in current revisions for *DSM*-V, through the work of Rosemary Basson, and Lori Brotto, who both suggest that female sexual response is more responsive and receptive, and less spontaneous, than male desire (Basson, 2002; Brotto, 2010).

4. Initial attempts to develop vasocongestive targeting arousal problems proved disappointing; efforts have since focused on ‘central pathways’ and the ‘desire centres’ of the brain, with testosterone and serotonin receptor antagonists candidates (e.g. flibanserin). The latter garnered much media attention, but was rejected by the FDA, which has not yet approved a pharmacological agent for FSD, though the European Medicines Agency has licensed Intrinsa, a testosterone patch for post-menopausal women with Hypoactive Sexual Desire Disorder (HSDD).

5. The critique features prominently in Moynihan and Mintzes’s recent popular book, making use of the tremendously fruitful language of ‘construction’, but in ways that slip between questioning the validity of the FSD category, and indeed of the very task of classification – an *epistemological* point – and questioning whether someone has symptoms that the diagnosis claims to describe – questioning whether there is anything there to (mis)describe – an *ontological* point. This obfuscation in the use of the term is where interested parties – pharmaceutical companies, practitioners invested in the dissemination of FSD and treatments for it – have immense leeway to accuse FSD critics of disregard for women. See Angel (2012).

6.
Downing (2004) stands out somewhat in that it uses critical and queer theory to approach FSD.
